# Normothermia for Pediatric and Congenital Heart Surgery: An Expanded Horizon

**DOI:** 10.3389/fped.2015.00023

**Published:** 2015-04-28

**Authors:** Ahmad Mahir Shamsuddin, Ahmad Mohd Nikman, Saedah Ali, Mohd Rizal Mohd Zain, Abdul Rahim Wong, Antonio Francesco Corno

**Affiliations:** ^1^Pediatric and Congenital Cardiac Surgery Unit, Department of Surgery, School of Medical Sciences, Universiti Sains Malaysia, Kubang Kerian, Kelantan, Malaysia; ^2^Department of Anesthesia, School of Medical Sciences, Universiti Sains Malaysia, Kubang Kerian, Kelantan, Malaysia; ^3^Department of Pediatrics, School of Medical Sciences, Universiti Sains Malaysia, Kubang Kerian, Kelantan, Malaysia; ^4^Pediatric Cardiology, Hospital Raja Perempuan Zainab II, Kota Bharu, Kelantan, Malaysia

**Keywords:** cardiopulmonary bypass, congenital heart defects, congenital and pediatric heart surgery, hemodilution, high flow, modified ultrafiltration, normothermia, surgical outcomes

## Abstract

Cardiopulmonary bypass (CPB) in pediatric cardiac surgery is generally performed with hypothermia, flow reduction and hemodilution. From October 2013 to December 2014, 55 patients, median age 6 years (range 2 months to 52 years), median weight 18.5 kg (range 3.2–57 kg), underwent surgery with normothermic high flow CPB in a new unit. There were no early or late deaths. Fifty patients (90.9%) were extubated within 3 h, 3 (5.5%) within 24 h, and 2 (3.6%) within 48 h. Twenty-four patients (43.6%) did not require inotropic support, 31 (56.4%) received dopamine or dobutamine: 21 ≤5 mcg/kg/min, 8 5–10 mcg/kg/min, and 2 >10 mcg/kg/min. Two patients (6.5%) required noradrenaline 0.05–0.1 mcg/kg/min. On arrival to ICU and after 3 and 6 h and 8:00 a.m. the next morning, mean lactate levels were 1.9 ± 09, 2.0 ± 1.2, 1.6 ± 0.8, and 1.4 ± 0.7 mmol/L (0.6–5.2 mmol/L), respectively. From arrival to ICU to 8:00 a.m. the next morning mean urine output was 3.8 ± 1.5 mL/kg/h (0.7–7.6 mL/kg/h), and mean chest drainage was 0.6 ± 0.5 mL/kg/h (0.1–2.3 mL/kg/h). Mean ICU and hospital stay were 2.7 ± 1.4 days (2–8 days) and 7.2 ± 2.2 days (4–15 days), respectively. In conclusion, normothermic high flow CPB allows pediatric and congenital heart surgery with favorable outcomes even in a new unit. The immediate post-operative period is characterized by low requirement for inotropic and respiratory support, low lactate production, adequate urine output, minimal drainage from the chest drains, short ICU, and hospital stay.

## Introduction

Cardiopulmonary bypass (CPB) for pediatric cardiac surgery is generally performed with hypothermia, flow reduction, and hemodilution. The reasons historically given to justify the above technique were to maintain low metabolic status during the operation, to gain maximum tissue protection during CPB, to have a relatively safe margin in situation of unexpected difficulties, and to provide adequate surgical exposure (1).

A large number of hospitals are still using the technique of deep hypothermia with circulatory arrest, justified by reduced duration of CPB in small infants, simplified cannulation and unencumbered operative field particularly for infants with anomalous venous connections (2).

However, the use of hypothermic CPB with flow reduction and hemodilution is associated with major side effects, with negative influence on the patients’ outcomes. Because of this reason several hospitals, at least in Europe, have moved toward the use of normothermia for pediatric and congenital heart surgery (1, 3, 4).

In a new unit like ours, with its inherent difficulties because of new staff and the lack of training available in a developing country, normothermic high flow CPB was introduced for pediatric and congenital heart surgery.

The aim of this prospective study was to analyze the results of the initial experience.

## Materials and Methods

From October 2013 to December 2014, 97 consecutive patients, median age 3.6 years (range 27 days to 52 years), with median body weight 12.1 kg (range 800 g to 62 kg), underwent surgery for pediatric and congenital heart defects in our new unit.

Criteria for exclusion from this study were operation performed without the use of CPB or operation performed with the use of even a mild degree of hypothermia (<35°C).

Fifty-five consecutive patients (55/97 = 57%), median age 6.0 years (range 2 months to 52 years), with median body weight 18.5 kg (range 3.2–57.0 kg), underwent surgery with normothermic high flow CPB and were prospectively included in the study.

Forty-two patients (42/97 = 43%) were excluded from the study because they were operated on either without CPB (40 patients) or because the operation required CPB with a period of hypothermia (<35°C) because of aortic surgery with flow reduction (2 patients).

The study was approved by the hospital ethical committee and parental informed consent was obtained for all patients.

### CPB protocol

The standard protocol for CPB was the following:

- priming with leukocyte-depleted blood (when blood required)
- controlled reoxygenation (5–9) in cyanotic patients with pre-operative oxygen saturation ≤85%
- normothermia: nasopharynx and rectal temperature maintained between 35.0 and 36.5°C
- high flow: ≥3.0 L/m^2^ BSA/min
- hematocrit ≥30%, aiming at a value at least 40% by the end of CPB
- mixed venous oxygen saturation ≥65%
- cold blood cardioplegia administered every 20 min
- modified ultrafiltration (10–15 mL/kg) at the end of CPB
- administration of I.V. milrinone (0.3 mcg/kg/min) from the end of CPB
- administration of I.V. calcium gluconate (300 mg/kg/day) on arrival to ICU
- administration of I.V. frusemide (1–2 mg/kg/day) on arrival to ICU

Diagnoses and the surgical procedures are listed in Tables 1 and 2.

**Table 1:** **List of diagnosis and associated defects**.

Number of cases	Diagnosis	Associated defects
24	Ventricular septal defects	9 AoV Regurgitation
	18 DCJA	7 RVOTO
	2 Perimembranous	7 Subaortic obstruction
	4 Multiple VSDs	
8	Atrial septal defect	4 MV Regurgitation
		1 Borderline LV
		1 Dislodged occlusion
		device
4	MV regurgitation	3 AoV regurgitation
		1 TV regurgitation
5	Tetralogy of Fallot	
4	AVSD	3 partial
		1 Complete
3	Anomalous pulmonary venous connection	2 partial
		1 Total
2	Obstructed RV-PA conduit	
1	RVOTO	s/p TF repair elsewhere
1	DORV, VSD, TGA, PS	
1	Mitral atresia	
	DORV	
	Hypoplastic LV	
	Restrictive inter-atrial communication	
1	Situs inversus	
	Dextrocardia	
	Univentricular Heart	
	Bilateral superior vena cava	
	Right aortic arch	
1	DORV, severe PS, VSD, hypoplastic PAs, right aortic arch	

*AoV, aortic valve; ASD, atrial septal defect; AVSD, atrio-ventricular septal defect, DCJA, doubly committed juxta-arterial, DORV, double outlet right ventricle, LV, left ventricle, MV, mitral valve, PA, pulmonary artery; PS, pulmonary stenosis; RV, right ventricle; RVOTO, right ventricular outflow tract obstruction; TGA, transposed great arteries; TV, tricuspid valve; TF, Tetralogy of Fallot; VSD, ventricular septal defect*.

**Table 2:** **List of surgical procedures**.

Number of procedures	Surgical procedures	Additional procedures
24	VSD closure	7 RVOT reconstruction
		7 Subaortic resection with septal myectomy
		2 AoV repair
8	ASD closure	4 MV repair
		1 Device removal
4	MV repair	1 MV replacement
		1 AoV repair
		1 TV repair
5	TF repair	1 RV–PA conduit
4	AVSD repair	
3	Repair of anomalous pulmonary venous connection	
2	Conduit replacement	
1	RVOT reconstruction with RV-PA conduit	
1	Intracardiac repair of DORV, VSD, TGA, PS	
1	Atrioseptectomy, main PA division, Bidirectional Glenn	
1	Atrioseptectomy, main PA division, bilateral bidirectional Glenn	
1	modified Blalock-Taussig shunt on CPB	

*AoV, aortic valve; ASD, atrial septal defect; AVSD, atrio-ventricular septal defect; CPB, cardiopulmonary bypass; DORV, double outlet right ventricle; MV, mitral valve; PA, pulmonary artery; PS, pulmonary stenosis; RV, right ventricle; RVOT, right ventricular outflow tract; TF, Tetralogy of Fallot; TGA, transposed great arteries; TV, tricuspid valve; VSD, ventricular septal defect*.

The Basic and Comprehensive Aristotle Score (10) were used to assess the potential mortality and morbidity in our patient population.

### Data collection

All database regarding patients operated with normothermic CPB were prospectively recorded.

The overall outcomes of the operation included: survival, need for re-operation, ICU, and hospital stays.

Intra-operative data included duration of CPB and aortic cross-clamp, need for inotropic support, lactate level, and urine output.

Post-operative data included duration of mechanical ventilation, and type, dosage and duration of inotropic support; in addition lactate level, urine output, and chest drains bleeding were recorded on arrival to ICU, after 3 and 6 h, and at 8:00 a.m. of the first post-operative day.

Our hospital does not have an intermediate step-down unit, and therefore the patients were discharged from ICU only when they were off ventilatory support, inotropes, and chest drains, and were transferred to the normal ward.

Data were expressed as mean ± SD.

## Results

The mean Basic Aristotle score (scale 1–5) was 3.1 ± 0.8 (range 1–5) and the mean Comprehensive Aristotle score (scale 1–10) was 6.2 ± 1.7 (range 3–10).

There were no early or late deaths until the end of the follow-up period (December 2014).

Not one patient had clinically evident neurological deficit, and only one patient (1/55 = 1.8%) with Down syndrome required re-operation because of wound infection caused by *Staphylococcus aureus* after repair of Tetralogy of Fallot.

Mean CPB time and aortic cross-clamp time were respectively 94 ± 43 min (range 45–288 min) and 46 ± 24 min (range 0–102 min).

Fifty patients (50/55 = 90.9%) were weaned from mechanical ventilation and extubated within 3 h from ICU arrival, three (3/55 = 5.5%) within 24 h, and two (2/55 = 3.6%) within 48 h.

Twenty-four patients (24/55 = 43.6%) did not require inotropic support. Thirty-one patients (31/55 = 56.4%) received dopamine or dobutamine post-operatively: 21 of them (21/31 = 67.7%) received dopamine or dobutamine ≤5 mcg/kg/min, 8 (8/31 = 25.8%) received 5–10 mcg/kg/min, and 2 (2/31 = 6.5%) required >10 mcg/kg/min. Two patients (2/31 = 6.5%) required additional noradrenaline 0.05–0.1 mcg/kg/min.

Mean lactate level at arrival to ICU, after 3 h, after 6 h and 08:00 a.m. next morning was respectively 1.9 ± 09, 2.0 ± 1.2, 1.6 ± 0.8, and 1.4 ± 0.7 mmol/L (range 0.6–5.2 mmol/L).

Mean urine output from ICU arrival to 08:00 a.m. next morning was 3.8 ± 1.5 mL/kg/h (range 0.7–7.6 mL/kg/h).

Mean chest drains bleeding from ICU arrival to 08:00 a.m. next morning was 0.6 ± 0.5 mL/kg/h (range 0.1–2.3 mL/kg/h).

Mean ICU and hospital stays were respectively 2.7 ± 1.4 days (range 2–8 days) and 7.2 ± 2.2 days (range 4–15 days).

## Discussion

Hypothermic CPB with hemodilution was introduced in pediatric cardiac surgery with the aims of decreasing the oxygen consumption and improving the distal body perfusion with the decreased blood viscosity due to hemodilution (1, 3, 4). With the introduction of surgical repair of more complex congenital heart defects in small infants, the hypothermic CBP with flow reduction, and even deep hypothermia with circulatory arrest, gained widespread application because of the adequate surgical exposure allowed by the absence of blood and venous cannulas in the small operative field (1, 2, 4, 11, 12).

Despite the good results obtained with hypothermic CPB, allowing the surgical repair of more complex congenital heart defects, over the years a large series of experimental and clinical studies reported extensive negative effects of hypothermia and flow reduction associated with hemodilution (1, 3, 4, 12–34), which are the following:

### At the cellular level

Decreased ATP levels were observed, as well as increased anaerobic metabolism, decreased intracellular pH, increased lactate production, decreased glycogen level, decreased efficiency of membrane-based ion pumps, increased cell swelling, decreased mitochondrial function, increased Calcium influx, and decreased intracellular enzyme function.

These damages mainly occur in the parenchymal cells (neurons and myocytes), the endothelial cells (systemic and pulmonary vascular systems), and the inflammatory cells (inflammatory response, ischemia/reperfusion injury).

### At the tissue level

The metabolic and hormonal systems are affected in relation to blood glucose, adrenal stress response, level of circulating adrenaline and noradrenaline, release of insulin and peripheral utilization of glucose, serum potassium, release of neurotransmitters in response to ischemia, ability of receptors to bind and take up noradrenaline, complement activation, release of angiotensin, interleukins, cytokines, beta-endorphines, and anti-diuretic hormones. The vascular system is affected by endothelial injury, decreased cardiac output, increased systemic vascular resistance, renal vasoconstriction, and generalized tissue edema.

In the myocardium, rapid cooling contracture can occur, and the respiratory system can be affected by endothelial lung injury.

Renal function is impaired by decreased glomerular filtration, decreased renal cortical blood flow because of renal vasoconstriction with redistribution of intra-renal blood flow to the renal medulla, and depressed tubular function.

Neurologic damages were reported because of increased cerebral vascular resistance, decreased cerebral blood flow, decreased response to increase in CO_2_ tension, hypothalamic dysfunction (post-operative hyperthermia), appearance of choreoathetosis, seizures, and overall vulnerability to brain injuries and neurodevelopmental impairment.

The hematologic system is affected by left shift of the oxyhemoglobin dissociation curve, leukocyte aggregation and degranulation, and platelet function defect (shape change, aggregation).

The overall clinical consequences complicating the outcomes of pediatric and congenital heart surgery with conventional hypothermic CPB with flow reduction and hemodilution are low cardiac output syndrome (requiring inotropic support), pulmonary dysfunction (requiring respiratory support), metabolic derangement (with acidosis and renal failure), coagulation derangement (with excessive chest bleeding), and neurologic complications (with choreoathetosis, seizures, and neurodevelopmental impairment) (1, 3, 4, 12, 15, 18–24).

All these negative reports derived from experimental and clinical studies motivated the search for alternative modality for perfusion in the pediatric population.

The two most important changes introduced in clinical practice were (1) the selective cerebral perfusion, in order to reduce the negative neurologic consequences of the circulatory arrest accompanying deep hypothermia (2, 11, 12, 35–39); (2) the reduced degree of hemodilution, with a higher hematocrit than used before (31, 33, 34).

But the most evident modification of the conventional CPB with hypothermia and hemodilution was the introduction of normothermic high flow CPB with minimal hemodilution.

This technique of perfusion was first used in Paris, France, by Lecompte and Durandy (40), who later reported in the literature the use of their technique in a very large number of patients in pediatric heart surgery (41, 42); the direct exposure to this experience persuaded other surgeons to introduce the same technique in their clinical practice, and in few years the number of hospital using normothermic high flow CPB with limited hemodilution expanded across Europe (1, 3, 4, 43–49).

The basic principles are the use of high flow, with the pump flow maintained ≥3.0 L/m^2^ BSA/min, with the nasopharynx and rectal temperature maintained between 35.0 and 36.5°C, and hematocrit maintained ≥30%. These conditions are more close to the normal physiology, where the systemic flow is 3.0–5.5 L/m^2^ B.S.A./min, the temperature 37°C, and the hematocrit 45%.

The pump flow used in conventional CBP is 2.0–2.4 L/m^2^ BSA/min or 100–120 mL/min/kg of body weight, even if it is improperly called “full flow,” and frequently is further reduced during the central part of the operation when requested by the surgeon to facilitate the surgical exposure, or even to circulatory arrest with deep hypothermia.

The combination of flow reduction, hypothermia, and hemodilutaion are responsible for all the difficulties observed in the post-operative course after pediatric and congenital heart surgery.

Additional modifications used in our experience, in addition to the technique of normothermic high flow CPB with limited hemodilution, were:

- priming with leukocyte-depleted blood (when blood required) The use of leukocyte-depleted blood was long time ago demonstrated to be one of the most important variable to reduce the ischemia/reperfuson damage associated with the CPB and the mycardial ischemia required for intracardiac repair (50, 51).
- “controlled” reoxygenation
  The damaged induced by the hypoxia/reoxygenation injury were extensively studied with experimental and clinical studies (5–9, 52–61).
  The technique of “controlled” reoxygenation was introduced in the clinical practice after extensive animals’ studies. To reduce the oxygenation damage, the arterial pO_2_ at the beginning of CPB is slowly and progressively increased, reaching the full reoxygenation after 5 min of full flow. This technique was applied in our experience in all cyanotic patients with pre-operative oxygen saturation ≤85%.
- modified ultrafiltration

The technique of modified ultrafiltration was introduced as one of the methods to reduce the inflammatory response caused by the CPB (62–69).

The mechanisms used by modified ultrafiltration are the following: removal of significant amounts of inflammatory mediators (IL-1, IL-6, TNF-alpha, C3a, and C5a), removal of excessive body water, and reduction in the quantity of circulating endotoxins.

The observed clinical advantages provided by modified ultrafiltration are improved intrinsic left ventricular systolic function, increased systemic blood pressure, decreased pulmonary artery pressure, and decreased requirement for inotropic and respiratory support in the early post-operative period (62–73).

In our experience, we applied modified ultrafiltration at the end of CPB in all patients, removing an amount of fluids between 10 and 15 mL/kg according to the clinical needs.

This study with normothermic high flow CPB has resulted in favorable outcomes, even in our study population, represented by patients with generally poor conditions because of malnutrition, recurrent infections, and late referrals. Furthermore, the technique has been introduced in a new unit, without any previous experience in pediatric cardiac surgery, and with all limitations due to reduced manpower, limited resources, and a generalized low level of expertise in the field.

Nevertheless, our patients had good results, with generally short ICU and hospital stay, early extubation, low requirement of inotropic support, low lactate production, adequate urine output, and minimal drainage from the chest drains.

### Potential concerns

There are two potential concerns toward the use of normothermic, high flow, high hematocrit, CPB:

(a) reduced margin of safety against potential incidents on CPB
(b) inadequate surgical exposure.

However, the advantages provided by this perfusion technique in the post-operative recovery overcome by far the potential risk of incidents (44, 45), and adequate exposure even in small cyanotic neonates can be obtained with appropriate venous cannulation and left heart venting (1, 40, 43–45).

With regard to all the neurologic complications reported by the conventional CPB, the normothermic, high flow, high hematocrit, CPB has been used with reported contradictory neurologic results, from the safety in relationship to the neurodevelopmental status (49) to the observation with comparison of pre-operative with post-operative magnetic resonance imaging suggesting that normothermic perfusion is associated with few new lesions following the surgical treatment (73).

### Limitations of the study

The main limitation of this study is the absence of a control group.

So far, no systematic randomized control trial has been performed to support normothermic high flow CPB versus hypothermic CPB, but there is an increasing trend toward the use of normothermic CPB, and no major adverse effects have been reported by any series on normothermic CPB.

Randomized controlled clinical trials (70) are considered the gold standard (71) and would help define which of the two main techniques are superior; however, a prospective randomized clinical trial is not easily feasible because of several reasons:

(a) huge variability among the patient populations with congenital heart defects. For instance, the evaluation of the neurodevelopmental outcome (70) should take into consideration a substantial percentage of infants with pre-operative cerebral abnormalities (72)
(b) standardization of surgical procedures, with endless variable details, is very difficult to achieve
(c) minor or major differences in the post-operative values of inflammatory markers, neurological status, requirement and duration of inotropic and respiratory support, stay in ICU, etc., are dependent upon several variables in the patient management, all inter-related and inter-dependent, not necessarily correlated with the modality of CPB
(d) after having used the “normothermic, high flow, high hematocrit” CPB, and having seen the advantages over the conventional hypothermic techniques, with an extremely smooth and “physiologic” post-operative course, to consider for the patients a return to the hypothermic techniques has been considered by all surgeons as undesirable or unethical

## Conclusion

Normothermic high flow CPB, close to normal physiology, allows pediatric and congenital heart surgery with favorable outcomes. The immediate post-operative period is characterized by low requirement for inotropic and respiratory support, low lactate production, adequate urine output, minimal losses from chest drains, short ICU, and hospital stay.

This CPB technique was introduced successfully in a new pediatric cardiac service with previous or little experience in congenital heart disease surgery.

## Conflict of Interest Statement

The authors declare that the research was conducted in the absence of any commercial or financial relationships that could be construed as a potential conflict of interest.
